# Association of Firmicutes/Bacteroidetes Ratio with Body Mass Index in Korean Type 2 Diabetes Mellitus Patients

**DOI:** 10.3390/metabo14100518

**Published:** 2024-09-26

**Authors:** Kainat Ahmed, Ha-Neul Choi, Sung-Rae Cho, Jung-Eun Yim

**Affiliations:** 1 Interdisciplinary Program in Senior Human Ecology, Changwon National University, Changwon 51140, Republic of Korea; kainat2gikian@gmail.com; 2 Department of Food and Nutrition, Changwon National University, Changwon 51140, Republic of Korea; chntony@changwon.ac.kr; 3 Changwon Fatima Hospital, Department of Endocrinology, Changwon 51394, Republic of Korea; cho34507@naver.com

**Keywords:** obesity, gut microbiota, F/B ratio, diabetes, fatty liver, AST

## Abstract

Background: The gut microbiome, which is the collection of microorganisms living in the gastrointestinal tract, has been shown to play a significant role in the development of metabolic disorders such as obesity and type 2 diabetes mellitus (T2DM). Studies have found that the ratio of *Firmicutes* to *Bacteroidetes* (F/B) is higher in obese individuals compared to lean individuals and tends to decrease with weight loss. However, the relationship between the F/B ratio and T2DM in Korean individuals, with or without obesity, is not fully understood. Objective: The objective of this study is to compare the F/B ratios and metabolic profiles of lean and obese Korean individuals with T2DM. Methods: In this study, 36 individuals with type 2 diabetes mellitus (T2DM) were recruited and classified into four groups (I, II, III, and IV) based on their body mass index (BMI). Group I had a BMI of less than 23.0, group II had a BMI between 23.0 and 24.9, group III had a BMI between 25.0 and 29.9, and group IV had a BMI of 30 kg/m^2^ or greater. Fecal samples were collected from all participants and sent to Chunlab Inc. (located in Seoul, Republic of Korea) for analysis. The changes in the major microbial phyla within the samples were investigated using quantitative real-time PCR. The collected data were then statistically analyzed using the SPSS program. Results: The levels of triglycerides and alanine transaminase in group I were significantly lower than in the other three groups. The amount of Actinobacteria in group IV was the highest among all four groups. The ratio of *Firmicutes* to *Bacteroidetes* increased as BMI increased, and this ratio was positively correlated with AST activity. Conclusions: Our study showed that there is a correlation between the degree of obesity in individuals with diabetes and their gut microbiome. Additionally, the ratio of *Firmicutes* to *Bacteroidetes* (F/B ratio) may play a role in the metabolic effects of fatty liver disease, as it may contribute to obesity.

## 1. Introduction

The human gut hosts a complex, dynamic, and heterogeneous microbial community of approximately 1 trillion (10^14^) microorganisms, with around 70% of them residing in the gastrointestinal tract and performing numerous functions in metabolism, energy homeostasis, epithelial cell proliferation, and gut barrier function [1,2,3]. This microbial community lives in a symbiotic relationship with the host, and its diversity can vary between individuals depending on their diet, their environment, and the type of delivery they undergo [4,5,6]. The gut has been found to carry a 150-fold higher number of genes than the human genome and can synthesize proteins that are not normally synthesized by the human genome itself such as enzymes involved in the breakdown of otherwise indigestible dietary elements in the human gut such as cellulose, hemicellulose, pectin, and other non-degradable starches [7,8]. The gut microbiome not only assists the uptake of nutrients and energy from ingested food but also helps in the degradation of starch, fructose, mannose, sucrose, galactose, and butanoate, and it also metabolizes N-glycan chains through the production of numerous glycoside hydrolases and polysaccharide lyases [9]. Microbial metabolism of these polysaccharides leads to the development of short-chain fatty acids (SCFAs) that are taken up by the host via diffusion and represent 10% of daily calorie intake depending on the dietary profile of the host [10]. On that account, it is not surprising to find links between gut microbiota irregularities and other metabolic diseases in an individual [11]. Dysbiosis is a term related to the disruption in the normal functioning of the gut microbiota that leads to various gut-related diseases such as obesity, diabetes mellitus, and inflammatory bowel diseases [12] such as irritable bowel syndrome (IBS). According to the human microbiome project, the gut microbiome is dominated by five major phylas; phyla *Firmicutes*, *Bacteriodetes*, *Actinobacteria*, and *Verrucomicrobia* and the archeal phylum *Euyarchaeota* [13,14]. *Firmicutes* and *Bacteroidets* both are the most prevalent and dominant of all, with *bacteroidetes* forming up to 30% of the gut microbiome [15].

Studies show that environment, diet, genetic factors, and the use of antibiotics have a strong influence on the diversity and existence of gut microbiota [16]. It is important to maintain the diversity and balance of the gut microbiota as a decrease in gut microbial diversity is shown to be associated with several disease conditions such as IBS and colorectal cancer [17]. Studies on mice show that conventional mice had 42% more fat than germ-free (GF)-grown mice although the GF mice utilized 29% extra. After the GF mice were conventionalized, there was a 57% and 61% increase in overall fat and epididymal fat despite decreased chow intake [18]. This shows that specific gut microbial populations can have specified effects on weight gain and a specific microbial population can be targeted for therapeutic purposes.

These findings further provide insight into the association of altered microbial populations with obesity. In both mice and humans, *Firmicutes* and *Bacteroidetes* are the dominant gut microbial phyla, and studies have shown an association between lower B/F ratios and obesity [19]. Roux-en-Y gastric bypass (RYGB) results show that a decrease in *firmicutes* and an increase in *bacteriodetes* and gamma *proteobacteria* plus *verrucomicrobia* in human subjects suggest that change in the gut microbiome can cause weight gain [20].

Obesity is defined as excessive fat accumulation in the body due to an imbalance between energy intake and energy expenditure by the host in relation to its environment [21]. Obesity is a multifactorial disorder that leads to excessive fat accumulation in the body to an extent that can lead to short- or long-term effects on the health of an individual [22]. The frequency of obesity worldwide is increasing at a fast pace, making it a challenging issue for humankind to cope with nowadays. According to data provided by WHO in 2016, about 40% of women aged 18 or above were found to be overweight, whereas 15% of them were obese worldwide [23]. The National Center for Health Statistics reported twice the prevalence of severe obesity in women than in men, i.e., 11.5% and 6.9%, respectively [24]. The escalating trend of obesity in Korea, as well as in Western societies, nowadays is of serious concern [25]. Obesity is also linked to an increased risk of other metabolic diseases such as type 2 diabetes mellitus (T2DM) and heart diseases, cancer, hyperlipidemia, metabolic syndromes, hypertension (HTN), and depression without specific symptoms [26]. Studies show that factors such as diet, physical activity, and medication can lead to obesity-induced diabetes mellitus [27]. As the gut microbiota plays an active role in energy metabolism, alteration in its population and function, at least in part, can lead to the development of obesity. The gut and liver are closely connected through the gut–liver axis, a bidirectional communication system. Changes in gut microbiome composition can influence liver function and vice versa. An elevated F/B ratio, indicating a higher proportion of Firmicutes relative to Bacteroidetes, has been associated with NAFLD. Firmicutes are more efficient at extracting energy from the diet, which can contribute to weight gain and the development of fatty liver disease. Certain gut-derived metabolites, such as short-chain fatty acids (SCFAs) and bile acids, can influence liver function and inflammation. An imbalance in the gut microbiome may lead to the production of pro-inflammatory metabolites, which can contribute to the elevation of liver enzymes like AST and ALT. An altered gut microbiome, particularly an increased F/B ratio, has been linked to increased intestinal permeability, also known as “leaky gut”. This can allow bacterial lipopolysaccharides (endotoxins) to enter the bloodstream and reach the liver, triggering an inflammatory response and potentially contributing to elevated liver enzyme levels. The gut microbiome can influence the expression of genes involved in glucose and lipid metabolism, as well as the production of hormones and signaling molecules. These changes in the gut–liver axis can contribute to the development of metabolic disorders, such as insulin resistance and NAFLD, which are often associated with elevated liver enzyme levels.

Diabetes mellitus is one of the associated disorders that are linked with abnormal energy distribution and low-grade inflammation in the body in obese subjects [28]. The number of people with type 2 diabetes mellitus (T2DM) is growing quickly worldwide. Experts predict that by 2045, 629 million individuals between 20 and 79 years old will have this condition [29]. The human gut microbiome is predominantly composed of two major bacterial phyla, Firmicutes and Bacteroidetes, which together make up over 80% of the total microbial population [30]. These phyla are further divided into more than 100 distinct bacterial species. While the significance of the ratio between Firmicutes and Bacteroidetes (F/B ratio) remains a subject of debate, research conducted by Larsen et al. using real-time quantitative polymerase chain reaction (qPCR) has revealed intriguing findings [31]. Their study demonstrated that individuals with type 2 diabetes mellitus (DM) exhibit a significant reduction in the proportion of Firmicutes compared to healthy subjects [31]. Moreover, they observed a notable negative correlation between the F/B ratio and plasma glucose levels, suggesting a potential link between gut microbiota composition and glucose metabolism in diabetic patients. Previous research has highlighted the importance of butyrate, a compound produced when certain bacteria digest dietary fibers, in maintaining gut health and metabolic function [32]. Butyrate has been shown to strengthen the intestinal barrier, reduce inflammation, and enhance insulin sensitivity [33]. A key player in butyrate production is *Faecalibacterium prausnitzii*, a bacterial species belonging to the *Clostridium leptum* group within the Firmicutes phylum [34]. Notably, studies have observed a decrease in the abundance of *F. prausnitzii* in individuals with type 2 diabetes mellitus, suggesting a potential link between this bacterial species, butyrate production, and metabolic health [35]. Recent research has highlighted significant alterations in the gut microbiome of individuals with type 2 diabetes mellitus (DM). Notably, there is a decrease in Bifidobacterium species, which typically work synergistically with butyrate-producing bacteria through metabolic cross-feeding [36]. Additionally, emerging evidence suggests that *Akkermansia muciniphila* may play a protective role against type 2 DM by improving glucose tolerance and reducing adipose tissue inflammation [37]. These findings underscore the complex relationship between gut microbiota and metabolic health, as the microbiome appears to influence both glucose metabolism and energy homeostasis. When the delicate balance of this microbial ecosystem is disrupted, a condition known as dysbiosis occurs. This imbalance can lead to changes in the composition of gut microbiota, increased intestinal permeability, and elevated production of metabolic endotoxins. Research has shown that dysbiosis is implicated in the development of several health issues, including obesity and type 2 diabetes mellitus. Research increasingly shows that changes in the balance of microorganisms living in the gut play a significant role in how T2DM develops [38]. A study conducted by Hanie S Ejtahed et al. [39] shows that gut microbiota plays a critical role in the progression of prediabetic conditions, i.e., the development of insulin resistance, and that obese individuals have an altered composition of gut microbiota, particularly having an elevated F/B ratio. Altered intestinal microflora and inflammation are observed in obese subjects, serving as a major cause for the development of type 2 diabetes mellitus. It has been found earlier that decreasing the level of *firmicutes* in the gut will reduce insulin resistance and associated factors that lead to the development of type 2 diabetes [40]. The occurrence of type 2 diabetes can thus be influenced by the microbial community that resides in the intestines; thereby, the diabetes–microbe association can be manipulated and utilized for the management of diabetes mellitus.

Previous research has shown that there are notable differences in the gut microbiome composition between obese individuals who have type 2 diabetes and those who do not, suggesting a potential link between gut bacteria, body composition, and the development or progression of diabetes [41]. Gut microbiota imbalance can play a significant role in increasing obesity by an alteration in the F/B ratio. However, the association of the F/B ratio with obesity in Korean type 2 diabetic patients has not been fully investigated. Therefore, this study aimed to evaluate the association between an altered F/B ratio and obesity in Korean type 2 diabetic patients.

## 2. Materials and Methods

### 2.1. Subjects and Study Design

The data were collected from 36 individuals, both male and female, who were type 2 diabetic during the period from May to November 2017. These subjects were divided into four groups, I, II, III, and IV, because of their BMI. Different cut points were used for body mass index (BMI) in this study due to the specific guidelines provided by the Western Pacific Region and the Korean Society for the Study of Obesity (KSSO). These organizations have established BMI thresholds that differ from the standard World Health Organization (WHO) classifications, primarily to better reflect the unique health risks associated with body weight in Asian populations. The groups were those with a BMI < 23.0 kg/m^2^ (lean weight persons), those with a BMI between 23 and 24.9 kg/m^2^ (overweight persons), those with a BMI between 25.0 and 29.9 kg/m^2^ (obese persons), and those with a BMI ≥ 30.0 kg/m^2^ (morbidly obese persons). Exclusion criteria for the patients were based on a history of diseases like anorexia, cancer, psychiatric disorders, and endocrinology diseases. This study was conducted at Changwon Fatima Hospital under the IRB number 17-04.

### 2.2. Anthropometry and Body Composition

Diabetes was defined based on blood glucose levels using American Diabetes Association criteria, self-reported diabetes history, or use of anti-diabetic medications. The information collected included demographic information, smoking and drinking habits, and clinical data from patient interviews and medical records at the time of enrollment. Hypertension was identified by either a reported history or use of anti-hypertensive drugs. This study also gathered information on medications, such as anti-diabetic agents, statins, and anti-hypertensive drugs, from medical records at enrollment (metformin, glipizide and glyburide, sitagliptin and canagliflozin). Body mass index (BMI) was calculated using the standard formula of weight in kilograms divided by height in meters squared. Anthropometric parameters including height, weight, waist circumference (WC), and hip circumference (HC) were measured for all subjects. Height and weight measurements were taken using a digital scale. The BMI for each subject was calculated by dividing the subject’s weight (kg) by the square of subject’s height (m^2^). Body fat mass (BFM) was measured using bioimpedance analysis (InBody 3.0; Biospace, Seoul, Republic of Korea). A flexible and substantial tape was used to measure the WC and HC. The waist-to-hip ratio (WHR) was calculated by dividing the WC by the HC.

### 2.3. Nutritional Intake Analysis

An analysis of the participants’ food intakes was performed for all groups. Subjects were assigned diet diaries to record all snack and meal consumption for a total of 3 days, including 2 weekdays and 1 weekend day. Before the diet recording was started, a registered dietitian equipped the subjects with the necessary information regarding the use of food diaries and recordings of eatables. Once the recorded information was obtained, an interview with the subjects was conducted to clarify the exact amount and type of food consumption. The collected data were then subjected to a computer-aided nutritional analysis program used by professionals for nutritional analysis (CAN pro version 5.0, Korean Nutrition Society). The nutritional intakes including energy, carbohydrate, protein, fat, fiber, and cholesterol content in each group for all subjects were determined.

### 2.4. Biochemical Analysis

For all groups, biochemical parameters including glucose, insulin, triglyceride (TG), total cholesterol (TC), high-density lipoprotein cholesterol (HDL-C), low-density lipoprotein cholesterol (HDL-C), alanine transaminase (ALT), and aspartate transaminase (AST) were measured. Plasma glucose, TG, TC, HDL-C, ALT, and AST concentrations were determined using commercially available kits based on enzymatic methods (Asan Pharm. Co., Seoul, Republic of Korea). Plasma insulin was measured using an enzyme-linked immunosorbent assay (ELISA) test kit (Invitrogen Thermo fisher scientific, Waltham, MA, USA). HbA1c levels were analyzed using the EASY A1c (Infopia Co., Anyang, Republic of Korea). Serum LDL-C content was calculated using the Friedewald formula: LDL-C = TC(HDL-C + TG/5. The results were then expressed as mg/dl of serum. Homeostasis model assessment was used to determine insulin resistance (HOMA-IR) using the formula HOMA-IR= [Fasting plasma insulin (µU/mL) × Fasting plasma glucose (mmol/L)

The atherogenic index (AI) was calculated using the formula: [(Total-C − HDL-C)/HDL-C].

### 2.5. Sample Collection and DNA Extraction

Fresh stool samples from each subject were collected on-site. Within 10 min after defecation, the fecal sample was aliquoted, and aliquots were stored at −20 °C for one day and then were immediately sent to Chunlab Inc. (Seoul, Republic of Korea) for analysis. The process involved weighing 50–100 mg of each sample, followed by a brief bead-beating step. Subsequent extraction steps adhered to the manufacturer’s protocol. DNA concentration and quality were assessed using a Colibri Microvolume spectrophotometer. Total DNA was extracted from samples using the Fast DNA SPIN Kit for soil (MP Biomedicals, Irvine, CA, USA), in accordance with the manufacturer’s instruction. The extracted DNA samples were promptly stored at −20 °C until further use.

### 2.6. PCR Amplification and NGS Sequencing

PCR amplification was performed using fusion primers targeting the V3 to V4 regions of the 16S rRNA gene with the extracted DNA. For bacterial genome amplification, fusion primers of 341F (5′-AATGATACGGCGACCACCGAGATCTACAC-TCGTCGGCAGCGTC-AGATGTGTATAAGAGACAG-CCTACGGGNGGCWGCAG-3′; underlining sequence indicates the target region primer) and 805R (5′- CAAGCAGAAGACGGCATACGAGATGTCTCGTGGGCTCGG-AGATGTGTATAAGAGACAG-GACTACHVGGGTATCTAATCC-3′). The fusion primers were constructed in an order with P5(P7) graft binding, i5 (i7) index, Nextera consensus, a sequencing adaptor, and a target region sequence. The amplifications were carried out under the following conditions: initial denaturation at 95 °C for 3 min, followed by 25 cycles of denaturation at 95 °C for 30 s, primer annealing at 55 °C for 30 s, and extension at 72 °C for 30 s, with a final elongation at 72 °C for 5 min. The PCR product was confirmed by using 1% agarose gel electrophoresis and visualized under a Gel Doc system (BioRad, Hercules, CA, USA). The amplified products were purified with Clean PCR (CleanNA). Equal concentrations of purified products were pooled together, and short fragments (non-target products) were removed with Clean PCR (Clean NA). The quality and product size were assessed on a Bioanalyzer 2100 (Agilent, Palo Alto, CA, USA) using a DNA 7500 chip. Mixed amplicons were pooled, and the sequencing was carried out at Chunlab Inc. (Seoul, Republic of Korea) with an Illumina MiSeq Sequencing system (Illumina, San Diego, CA, USA) and a standard kit (Kapa Biosystems, Wilmington, MA, USA) according to the manufacturer’s instructions.

### 2.7. Statistical Analysis

Statistical analysis was performed using the IBM SPSS program version 23. Values were expressed as mean ± standard deviation (SD). The differences among the groups were evaluated with a one-way ANOVA test followed by a Duncan post hoc test, and a *p*-value < 0.05 was considered statistically significant. The correlations between the *Fermicutes*/*Bacteroidetes* ratio, BMI, and serum AST and ALT values were analyzed by Spearman’s correlation test. Correlation was determined based on the correlation coefficient (r value) and considered significant if the *p*-value was < 0.05.

## 3. Results

### 3.1. General Characteristic and Anthropometric Parameters

The mean age of the subjects was 45 years, with a total of 24 males and 12 females. The general characteristics and anthropometric parameters of the subjects are shown in Table 1. The body weight, body mass index (BMI), body fat mass (BFM), and waist circumference (WC) of group IV were found to be significantly higher than those of the other groups. The body weight was found to be significantly different between all groups, and the highest value was observed in group IV. The BMI value was found to be significantly different between groups I and IV, whereas groups II and III showed no significant difference between them. The BFM value for groups IV and III was found to be significantly different from other groups, whereas the least significance was observed between groups I and II. The WHR values of the group IV and group I subjects were significantly higher than those of other groups. However, no significant difference in WHR was observed between groups II and III.

### 3.2. Nutritional Intakes

Data on daily nutrient intake are presented in Table 2. The caloric intake of group IV was higher than that of the other groups. However, no significant difference was observed among the other three groups. In group II, the amount of carbohydrate intake was high, but the difference was not significant. The amount of protein and fat intake in group IV was higher and significantly different from other groups. The fiber intake was found to be higher in group II but not significantly different compared to other groups. The cholesterol intake was high in group IV but not significantly different from other groups.

### 3.3. Glycemic Indices and Lipid Profiles

The parameters of the glycemic indices and lipid profiles are shown in Table 3. The plasma glucose levels of groups I, II, III, and IV were not significantly different. However, the serum insulin levels of group II were significantly higher than those of other groups. Serum total cholesterol did not significantly differ among all the groups. Although the HDL-C and LDL-C levels did not significantly differ among the groups, the serum TG level of group II was the highest, while that of group I was the lowest. The atherogenic index value was not significantly different among the groups. However, the serum ALT activity was significantly high in groups II, III, and IV, whereas it was the lowest in group I. The serum AST value was not significantly different among all the groups.

### 3.4. The Firmicutes/Bacteroidetes Ratio

The composition of the bacterial microbiome at the phylum level is shown in Table 4. The *bacteroidete* content was highest in group II compared to other groups. However, no significant difference in *bacteroidete* and *fermicutes* was observed among all the groups. In accordance with previous studies, our results also showed that the F/B ratio was increased with an increase in BMI. The maximum increase in the F/B ratio was observed for group IV.

**Figure 1:**
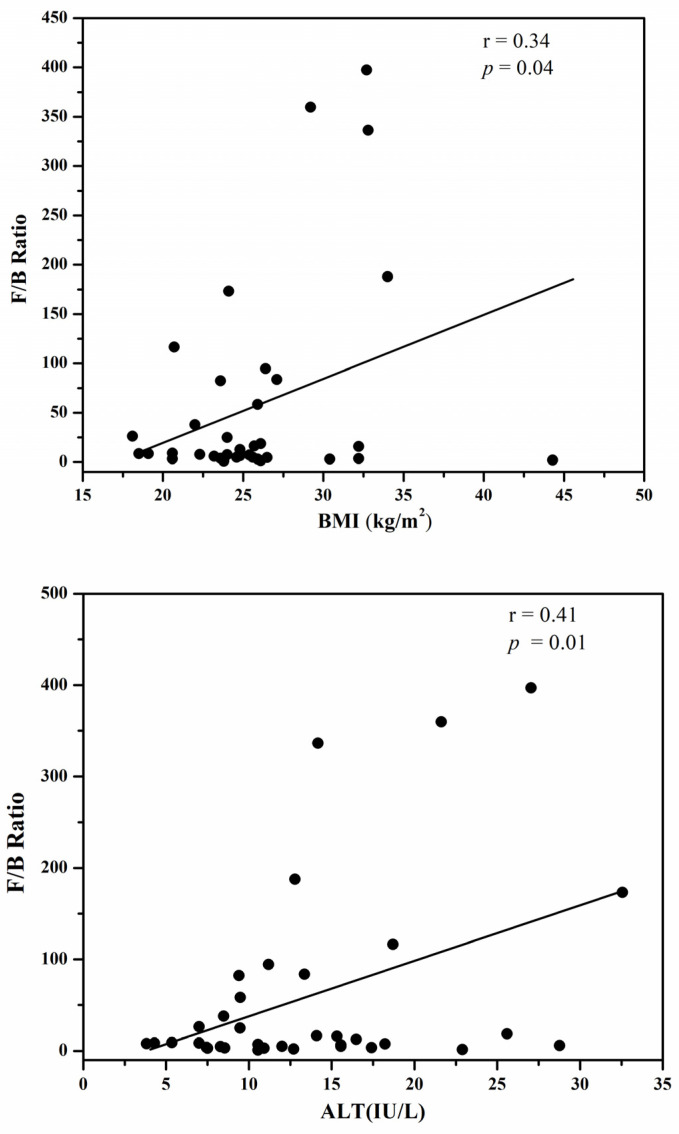

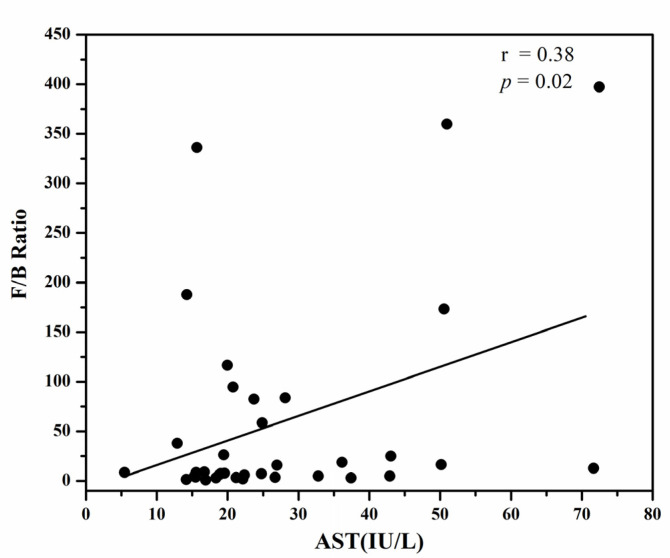
The positive correlation of the F/B ration with BMI (Body Mass Index), serum ALT (Alanine transaminase) and serum AST (Aspartate aminotransferase).

The F/B ratio was found to be positively correlated with the serum AST in obese Korean type 2 diabetic patients, as shown in Figure 1. The *r* value of 0.38 showed that the serum AST levels correlated positively with the F/B ratio; as a higher serum AST content is associated with fatty liver disease in an obese state, it can be predicted that the F/B ratio, because of its possible contribution to obesity, can imply the metabolic consequences of hepatic steatosis.

## 4. Discussion

The prevalence of obesity in the past few decades has been increasing at an alarming rate worldwide [42]. According to an WHO report, about 13% of the world’s adult population was obese in the year 2016 [43]. Thus, obesity has become a major public health concern worldwide and has resulted in many other obesity-related diseases such as diabetes, cardiovascular diseases, cancer, hyperlipidemia, metabolic syndromes, hypertension, and depression. We investigated the association of the ratio of the gut-microbiome-dominating microbes *firmicutes* and *bacteroidetes* with obesity in Korean type 2 diabetic patients. Consistent with the other studies, our results also showed a significantly higher F/B ratio in obese type 2 diabetic Korean patients.

In recent years, the significance of gut microbiota for health has been highly acknowledged. Recent studies showing a significant association between the gut microbiome and obesity have further emphasized the role of the gut microbiome [44]. Our study depicted consistent results with the previous studies showing an increase in the F/B ratio with an increase in obesity. Some studies reveal that instead of an increase in the F/B ratio, the trend of obesity is correlated with an increase in *bacteroidetes* content only [15]. This difference can be explained by many other factors like dietary intake, environmental conditions, physical activities, and socio-economic impacts. The *Actinobacteria* phylum, which consists mainly of the *bifidobacterium* genus, has also been found to be associated with weight gain and obesity. Previously researchers have observed higher levels of *actinobacteria* in obese subjects compared to lean [45]. Our study was consistent with similar results showing an increased *actinobacteria* content in obese diabetic patients compared to lean. Serum enzymes such as aspartate aminotransferase, alanine aminotransferase, lactate dehydrogenase, alkaline phosphatase, and ᵞ-glutamyltransferases are used as markers for evaluating liver function.

There is a complex relationship between biological markers such as aspartate aminotransferase (AST) and alanine aminotransferase (ALT), the gut microbiome, and obesity. Elevated levels of AST and ALT, enzymes commonly associated with liver function, are often observed in individuals with obesity, reflecting liver stress or damage, particularly non-alcoholic fatty liver disease (NAFLD). The gut microbiome plays a crucial role in modulating metabolic processes, influencing inflammation, and regulating energy balance, all of which are key factors in obesity. Dysbiosis, or an imbalance in the gut microbiome, can exacerbate liver inflammation and increase AST and ALT levels. This imbalance may contribute to the development of obesity and related metabolic disorders by affecting gut barrier integrity, promoting endotoxemia, and altering lipid metabolism. Therefore, the interplay between AST, ALT, the gut microbiome, and obesity highlights the importance of a healthy gut in maintaining metabolic health and preventing obesity-related liver diseases. Elevated levels of aspartate aminotransferase (AST) in serum enzymes have been reported in cases of liver dysfunction. Recent studies have also found abnormal levels of hepatic enzymes and fatty liver disease in obese subjects [46]. Our study showed that the F/B ratio positively correlated with BMI together with serum AST and ALT levels, thereby predicting a possible contribution of the F/B ratio to hepatic steatosis in obese diabetic subjects. Our findings are in agreement with the previous studies, which demonstrated the strong correlation between BMI and serum hepatic enzymes such as AST, ALT, and GGT.

One limitation of our study was the relatively low sample size and the targeting of the analysis of stool samples rather than the small intestine and proximal gut which is the most appropriate part where most of the nutrient exchange takes place and hence is directly involved in the effect of gut microbiota on body mass and metabolic diseases. These limitations need to be addressed in the future. Further investigation of the association between BMI and the F/B ratio in the Korean diabetic population needs to be performed at a lower taxonomic level.

## 5. Conclusions

Our findings contribute to the growing evidence that gut microbiome composition may play a significant role in the development and progression of metabolic disorders, such as obesity and type 2 diabetes. These results suggest that targeted interventions aiming to modulate the gut microbiome, such as dietary modifications, prebiotic/probiotic supplementation, or fecal microbiota transplantation, could potentially be leveraged as adjunctive therapies or preventative strategies for these metabolic conditions. Further research is needed to establish the clinical utility of gut-microbiome-based biomarkers or therapeutic approaches, but our study provides additional support for the gut–metabolic axis as a promising target for clinical management.

## Figures and Tables

**Table 1:** General characteristics of the subjects.

	Groups			
	I (N = 8)	II (N = 10)	III (N = 11)	IV (N = 7)
Age (year)	44.8 ± 11.7	50.0 ± 8.6	43.3 ± 8.8	39.7 ± 12.4
Male (n, %)	3 (37.5)	7 (70.0)	8 (72.7)	6 (85.7)
Female (n, %)	5 (83.2)	3 (37.5)	3 (37.5)	1 (12.5)
BW (kg)	54.0 ± 6.2 ^a^	67.4 ± 6.6 ^b^	75.8 ± 8.7 ^b^	99.1 ± 19.4 ^c^
Height (cm)	163.1 ± 5.9	167.1 ± 7.5	169.3 ± 8.8	170.0 ± 8.1
BMI (kg/m^2^)	20.2 ± 1.5 ^a^	24.1 ± 0.5 ^b^	26.4 ± 1.1 ^b^	34.1 ± 4.6 ^c^
BFM (kg)	13.0 ± 2.8 ^a^	16.3 ± 2.2 ^ab^	22.3 ± 4.2 ^b^	38.1 ± 14.0 ^c^
Waist (cm)	74.1 ± 11.5 ^a^	84.4 ± 3.2 ^ab^	90.0 ± 5.2 ^ab^	106.2 ± 10.0 ^b^
Hip (cm)	85.2 ± 6.8 ^a^	93.5 ± 4.8 ^ab^	97.9 ± 4.4 ^ab^	109.4 ± 11.2 ^b^
WHR	0.91 ± 0.1	0.93 ± 0.0	0.96 ± 0.0	1.02 ± 0.0

The participants were classified into groups I, II, III, and IV based on the BMI values of <23.0, 23.0–24.9, 25.0–29.9, and ≥30 kg/m^2^, respectively. BMI, body mass index; BFM, body fat mass; WHR, waist and hip circumference ratio. Means in the same row not sharing a common letter are significantly different at *p* < 0.05. Letters a, b, and c show statistical significance between the values.

**Table 2:** Nutritional intakes of the subjects.

	Groups			
	I (N = 8)	II (N = 10)	III (N = 11)	IV (N = 7)
Energy (kcal)	1560.2 ±379.0	1665.0 ± 407.2	1557.2 ± 289.5	2026.5 ± 810.5
Carbohydrate (g)	208.9 ± 77.7	269.0 ± 65.6	228.2 ± 48.7	248.6 ± 115.5
Protein (g)	60.8 ± 4.2 ^a^	63.0 ± 6.4 ^a^	62.0 ± 4.9 ^a^	94.2 ± 13.6 ^b^
Fat (g)	51.9 ± 20.1 ^ab^	38.3 ± 16.6 ^a^	44.3 ± 14.0 ^a^	70.6 ± 32.6 ^b^
CHO/Pro/Fat (%)	52.5:16.0:31.2	64.9:15.2:20.7	58.8:15.8:25.4	48.9:19.1:31.7
Fiber (g)	15.8 ± 7.1	22.0 ± 7.5	17.7 ± 4.9	20.7 ± 11.0
Cholesterol (mg)	162.7 ± 80.6	203.7 ± 146.6	160.2 ± 106.3	245.5 ± 107.3

The participants were classified into groups I, II, III, and IV based on the BMI values of <23.0, 23.0–24.9, 25.0–29.9, and ≥30 kg/m^2^, respectively. CHO, carbohydrate; Pro, protein. Means in the same row not sharing a common letter are significantly different at *p* < 0.05.

**Table 3:** Biochemical parameters.

	Groups			
	I (N = 8)	II (N = 10)	III (N = 11)	IV (N = 7)
Glucose (mg/dL)	129.4 ± 15.1	118.2 ± 5.9	134.2 ± 9.7	127.4 ± 13.2
Insulin (μlU/mL)	6.3 ± 1.2	9.8 ± 1.8	6.2 ± 0.8	5.6 ± 0.6
HbA1c (%)	7.7 ± 1.8	7.1 ± 0.7	7.5 ± 2.4	8.2 ± 2.1
HOMA-IR	2.1 ± 0.6	2.9 ± 0.6	1.9 ± 0.2	1.7 ± 0.3
TC (mg/dL)	180.7 ± 30.5	186.4 ± 26.6	158.3 ± 46.8	190.2 ± 20.9
TG (mg/dL)	59.5 ± 30.0 ^a^	215.5 ± 150.2 ^b^	185.7 ± 74.3 ^b^	168.8 ± 53.4 ^b^
HDL-C (mg/dL)	34.0 ± 11.1	35.0 ± 8.8	30.8 ± 12.4	30.0 ± 7.2
LDL-C (mg/dL)	55.5 ± 10.1	60.2 ± 9.9	49.7 ± 15.9	62.4 ± 12.7
Atherogenic index	4.7 ± 1.7	4.7 ± 1.8	4.5 ± 1.6	5.6 ± 1.4
ALT (IU/L)	7.9 ± 4.7 ^a^	16.4 ± 8.4 ^b^	14.2 ± 6.3 ^ab^	15.8 ± 5.4 ^b^
AST (IU/L)	16.3 ± 5.2	32.5 ± 18.7	28.8 ± 12.7	30.8 ± 20.0

The participants were classified into groups I, II, III, and IV groups based on the BMI values of <23.0, 23.0–24.9, 25.0–29.9, and ≥30 kg/m^2^, respectively. TC, total cholesterol; TG, triglyceride; HDL-C, high-density lipoprotein cholesterol; LDL-C, low-density lipoprotein cholesterol; ALT, alanine aminotransferase; AST, alanine transaminase. Means in the same row not sharing a common letter are significantly different at *p* < 0.05.

**Table 4:** Composition of the bacterial microbiome (phylum).

	Group			
	I (N = 8)	II (N = 10)	III (N = 11)	IV (N = 7)
Bacteroidetes (count)	4676.6 ± 4349.2	8357.3 ± 9130.2	5702.7± 6959.3	5805.3 ± 6666.1
Firmicutes (count)	43,055.9 ± 10,213.7	45,515.9 ± 10,314.0	42,069.9 ± 16,139.8	36,276.3 ± 9154.8
F/B ratio	27.1 ± 37.9	32.2 ± 55.1	59.3 ± 105.3	135.0 ± 172.6

The participants were classified into groups I, II, III, and IV groups on the BMI values of <23.0, 23.0–24.9, 25.0–29.9, and ≥30 kg/m^2^, respectively. F/B ratio, Firmicutes/Bacteroidetes ratio. Means in the same row not sharing a common letter are significantly different at *p* < 0.05.

## Data Availability

Data is contained within the article.
